# Exploring mentorship experiences among early career general dental practitioners in NHS dentistry

**DOI:** 10.1038/s41415-025-8721-4

**Published:** 2026-01-23

**Authors:** Pavni K. Lakhani, Hoda Wassif

**Affiliations:** 41415471251001 Cheshunt Community Hospital, Hertfordshire NHS Trust, UK; 41415471251002 University of Bedfordshire, Bedford, UK

## Abstract

**Introduction** This study focuses on exploring mentoring experiences of early career general dental practitioners who deliver care within the NHS (National Health Service).

**Aim** The aim of this study was to investigate experiences of mentorship interactions and the perceived value placed on mentoring within the profession.

**Methods** Empirical qualitative data were collected using semi-structured interviews of seven dentists who qualified within the past ten years, with an average of 45 minutes for each interview. The data were analysed using thematic analysis.

**Results** Four overarching themes were identified: the individual, the mentor, the process, and the environment.

**Conclusion** Participants valued mentoring and its influence on their professional life. They highlighted the importance of having a committed mentor, a supportive mentoring environment and a mentoring approach that is tailored to their individual needs. The findings of this small qualitative study raised questions around how the profession value mentoring and some of the challenges in quantifying such value.

## Key points

- Explores how newly qualified practitioners experience mentoring in practice.
- Highlights some of the challenges around how mentoring is viewed in the profession.
- Discusses ways to enhance the role of mentorship in dental practice.

## Introduction

Within the healthcare context, mentoring has been more focused on ‘the process whereby an experienced, highly regarded, empathetic individual, the mentor, guides another individual, the mentee, in the development and re-examination of their own ideas, learning, and personal and professional development'.^1^ For dental practitioners, this type of mentorship is included as part of the dental foundation training (foundation dentists: FD), or performers list validation by experience (PLVE) for overseas-qualified dentists.

Mentorship is carried out by the educational supervisor (ES) as part of those training schemes.

The discussion around mentoring and its role has been part of the recent debate focusing on approaches needed in supporting the development of the workforce.^2^^,^^3^ Mentoring has also been considered as a possible tool to help address some of the challenges of the steady decline in NHS (National Health Service) dentists,^4^ the increasing work pressures, and surging burnout rates.^5^ Additionally, existing literature in healthcare exhibits the benefits of mentoring on individuals relating to undergraduate medical students,^6^ as well as nurses.^7^ However, little insight has been provided into the influence of mentoring on the culture of dentistry or on individual general dental practitioners (GDPs) themselves, especially among early career practitioners. Furthermore, there are limited qualitative data surrounding the experiences of qualified dentists who have undertaken mentoring or their understanding of what mentoring involves. There seems to be a deficiency in the literature regarding the remits of an ideal mentorship or a mentor-mentee relationship in the context of dental practice.

This study seeks to add to the current literature and provide an insight into the views of early career dentists about their mentorship experiences. Gaining an understanding of the perceived value as well as the challenges of mentoring could potentially facilitate the development of future mentoring schemes, tailoring them to the needs of the GDPs and enhancing the current understanding of mentoring within dentistry.

## Background

McKimm and colleagues^8^ highlighted that mentoring could be an important medium for newly qualified practitioners assisting in their transition into practice and developing their confidence as independent and autonomous practitioners. This was echoed by others in the literature,^9^^,^^10^^,^^11^ where mentorship programmes were studied among undergraduate dental students and were found to have a positive influence on both the mentors and mentees' motivation and professional attitudes. While it could be argued that the undergraduate learning environment does not truly reflect the clinical work environment, these studies highlighted the potential value of mentoring for early career GDPs. There is also evidence that having mentors in the workplace contributes to an individual's sense of belonging, as well as connectedness to their profession.^12^

The literature highlighted the challenges of quantitatively evaluating the organisational benefit of the mentoring programme and return on investment (ROI).^13^ Others argued that ‘ROI is a poor measure of coaching success'.^14^ This small research study therefore sought to use a more qualitative approach to assess mentoring value in depth while recognising the need to quantify financial and organisational benefits.

Successful mentorships require engagement from both the mentors and the mentees, with appropriate training being provided in the roles and responsibilities as well as the scope of mentorship.^15^ Research identified the need for effective mentors in dentistry to display the characteristics of competence, confidence and commitment,^16^^,^^17^^,^^18^ with an emphasis that mentors should seek to understand the strengths of their mentees and should support them in both their professional and personal life. Both mentoring and coaching have similar requirements^19^ and while coaching and mentoring do vary in terms of the sports setting, the terms are often used interchangeably in the healthcare setting.

## Methods

The aim of this study was to explore the mentoring experiences of early career GDPs who provide NHS dental care using a qualitative approach.^20^^,^^21^ This study explored how participants experienced mentorship interactions, and the perceived value placed on mentoring within dental profession. Early career GDPs were defined as those who had qualified within the past ten years between 2013–2023 (inclusive).

Ethical considerations were made in accordance with the declaration of Helsinki and ethical approval was obtained from the University of Bedfordshire (Ref 01/DLE/02/2023 −2007880). Consent was also obtained from the NHS England (NHSE) East Midlands and West Midlands dental team to allow information about the study to be included in the local dental network (LDN) newsletters asking for GDPs to participate. This was in addition to invitations through professional social media forums. Participants were recruited using purposive sampling^22^ and semi-structured interviews^23^^,^^24^ were used for data collection.

All GDPs who met the inclusion criteria were eligible to participate in the study regardless of the country of graduation. An information sheet and consent forms were sent to all those who expressed interest in taking part. A total of nine participants responded to the invitation to take part. Thematic saturation was reached after six interviews and out of the nine participants who responded to the invitation, only seven (three women, four men) took part in the interviews which took place via Microsoft Teams. Two potential participants dropped out due to their availability. All participants completed their training in the United Kingdom apart from one who completed their training in Europe. Interview length ranged between 40–60 minutes with an average of 45 minutes. Each participant (P) was designated a number P (1–7) in order to maintain anonymity.

Data collected provided a manageable yet rich source of qualitative data. Interviews were conducted, recorded, transcribed and coded by the first author after training. Interviews were transcribed verbatim using MS Teams auto-transcription which was later checked for accuracy before anonymising the data. Transcribing the interviews formed the initial stages of the thematic analysis as it allowed the authors to immerse themselves in the raw data and to effectively observe any emerging themes. Manual coding took place, with themes and subthemes later identified throughout the data. Data were checked and reviewed by both authors at different stages of the process to ensure consistency, enhance credibility, and maintain accuracy in the interpretation of emerging themes.

A reflexive and reflective diary was kept in order to both prospectively and retrospectively create an awareness of potential external and internal influences on data interpretation. Factors such as professional roles and the ethics of insider researcher were considered as potential factors influencing the interpretation of data. Data were reviewed and checked by the authors independently in an ongoing process of evaluation to maintain objectivity and preventing bias.

## Results

Qualitative data were deconstructed with patterns and themes identified using Braun and Clarke's six-phase framework of thematic analysis using an interpretative paradigm and adopting an inductive approach to data analysis.^25^

The overarching themes were selected based on the frequency of their occurrence in the interviews and significance to the research question. Considerations were made in relation to the depth of discussion during the interviews in relation to the emerging themes.

Four emerging themes were identified: the individual, the mentor, the process, and the environment, with further associated subthemes.

### The individual

**Fig. 1:**
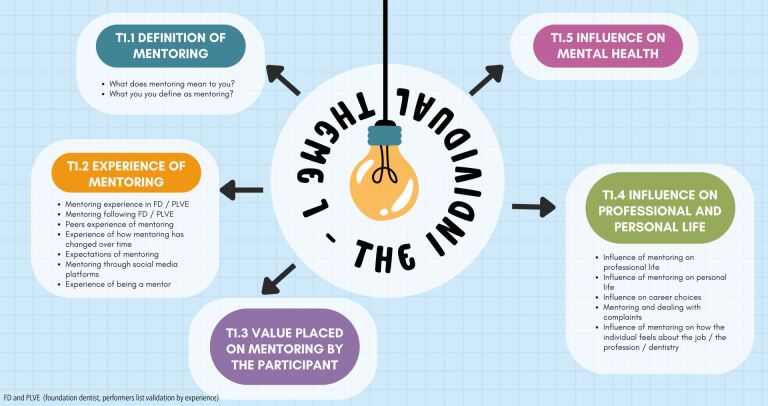
Theme 1: the individual

The perceived benefits of mentoring as a tool for developing and supporting the individual was reported by all participants. Subthemes are identified in Figure 1.

Participants shared their definition of mentoring which often included someone more experienced providing guidance and support:

- ‘*It means someone more experienced giving someone less experienced advice and guidance'* P1.

However, some participants challenged this notion:

- *‘…it doesn't have to be someone older…they can still give me guidance and support'* P7.

Participants also described their expectations, apprehensions and experiences of being mentored and, recognised the support it provided:

- ‘*I had mixed stories from my friends that had graduated before me. So I didn't really know what to expect to be honest*' P3.

Participants spoke about their own positive experiences:

- ‘*I would say I was quite lucky. I had a very good trainer…I learnt a lot…I got a good amount of clinical experience, but more than that I got a good amount of support*…' P7.

They also described the negative experience of peers:

- ‘…*I'm also aware that a lot of my friends who are the same year hated it and really struggled and now don't do general dentistry'* P2.

Some participants experienced the continuation of mentoring after the completion of their FD year in person and considered support through social media (e.g., WhatsApp) as mentoring:

- *‘…it is so helpful, because if someone can't be there in person [a mentor can be there via text]'* P2.

However, others stated that rather than mentoring, they felt there was a need for peer-to-peer support:

- *‘…[you will] get to a point where you are clinically able and clinically competent at what you do…do you need a formal mentoring programme? Probably not, but I do feel you need peer-to-peer support'* P5.

Some described the lack of support after completing the vocational training year:

- *‘…you're kind of just left to your own devices and again, that's where a lot of my friends have not enjoyed being an associate…*' P2.

The value placed on mentoring interaction was also explored:

- *‘…I definitely think that there's a value in mentorship because it's a profession where you're always learning and developing your skills…'* P1.

The influence of mentoring on personal and professional life including complaints and how GDPs feel about the profession was also a feature:

- ‘*She [my mentor] helped calm me as well because I got panicked as [it was] my first complaint…*' P3.

Only one participant felt that mentoring hadn't influenced their professional life:

- *‘I feel like mentoring hasn't had a huge impact on my professional [life]. Just due to the nature of our jobs'* P1.

The participant then went on to mention the isolating nature of the job.

Participants spoke about how mentoring had influenced how they felt about the profession and the job as well as other areas where they needed support:

- ‘*It [mentoring] made me like dentistry. I like coming to work. I like the patients that I've got. I like doing just general dentistry…'* P2
- *‘… in my personal life, they [mentors] helped me figure out how to pay my taxes, and what I needed to do, whether I want to be a company or self-employed, how to do all of that…*' P2.

Participants also spoke about the influence of mentoring on reducing their anxiety:

- *‘…a lot of other people probably have mental health breakdowns…one big thing I learnt from my mentor and I still learn from him is sort of compartmentalising them [work and personal life]…'* P7.

### The mentor

**Fig. 2:**
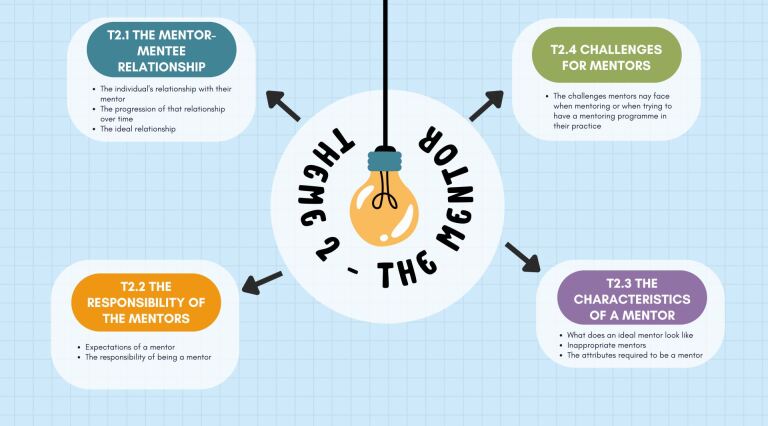
Theme 2: the mentor

‘The mentor' was another identified theme and Figure 2 outlines its content and subthemes.

Participants' views about the mentor and the relationship were outlined:

- *‘…I think once you finish university, you become more of a peer rather than kind of a student…'* P1
- ‘*Initially I thought a little bit like I was being babysat by someone, but that might have been my own ego because in the end it was quite nice…*' P3.

The differences in the mentor-mentee relationship were apparent throughout the interviews, with one participant stating that they intentionally chose a relaxed mentor:

- ‘…*he [the mentor] was very much laid back…my thought was, if I mess anything up, he's not gonna shout at me…*' P6.

The evolution of the mentor-mentee relationship was also described by participants:

- ‘*There came a point where it stopped being a mentee-mentor relationship and more of a casual friendship towards the end as in a group of peers…*' P5.

Participants spoke about their expectations of mentorship:

- *‘…they don't always need to kind of be there doing something, but they can just kind of be giving encouragement, like from afar…*' P1.

Three clear characteristics came through from the interviews regarding the character of a good mentor: willingness to allocate time to mentoring, skills to mentor, and patience.

Participants spoke about the need for mentors to ensure a reduced number of patients in their own clinical diaries to allow time for mentors to support the individuals:

- *‘… you've got to want to do it. You've got to have the skills to do it and you've got to have the patience because when you don't have all three, I don't think it works'* P2
- ‘*I think if you're…[going to] take on the role of a mentor, it's not a short-lived thing…I think if you're gonna cut them [the mentee] off, you're not doing mentoring for the right reason'* P6.

Participants also understood the challenges of being a mentor even if they had no formal experience of being one themselves:

- ‘*It [mentoring] does take a lot of time and energy'* P1
- *‘…you're not in control of their [the mentee's] skill level, so that that creates doubt in your mind and a bit of panic and stress'* P3.

### The process of mentoring

**Fig. 3:**
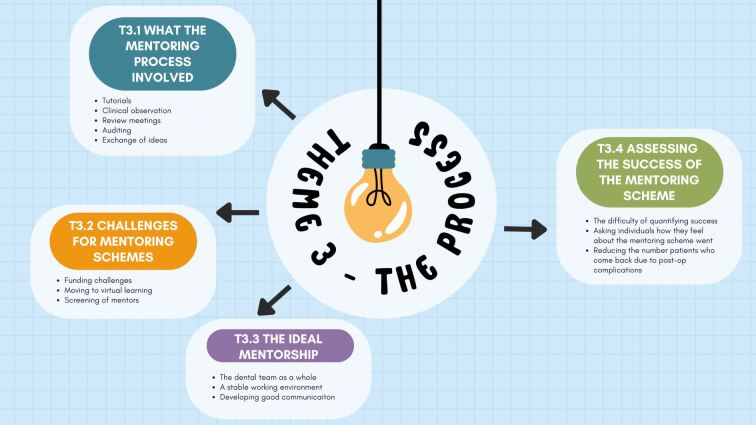
Theme 3: the process of mentorship

Figure 3 reflects the third theme its subthemes.

Participants expressed their understanding about the mentoring process itself:

- ‘*We used to have weekly kind of progress meetings and we would pick a topic that I felt like I wanted further help with and we would go through that topic…*' P1
- ‘*Once a week she [the mentor] would pick five random patients or ten and she would just look through my notes and she would leave me a note…'* P3.

The challenges of mentoring schemes were also considered:

- ‘[*there should be] better screening of ESs so that if someone is not suitable or the practice is not suitable, they're not going to get an FD who's going to really struggle…'* P2.

Participants also expressed a need for the mentoring process to be a more informal and tailored to the needs of the GDP:

- ‘*Certainly for me, something a bit more informal…friendlier-based situation, bit more casual discussion…'* P4
- ‘*I would say that as a dental graduate that's freshly out [of university], you need something that is somewhat structured and somewhat flexible…you need a helping hand that's there…*' P5.

All participants alluded to the difficulty of assessing the success of a mentoring programme:

- ‘*I don't think you can put it in figures and numbers. I think it's how someone feels that scheme has gone and what they find the value is in…*' P6.

### The environment for mentorship

**Fig. 4:**
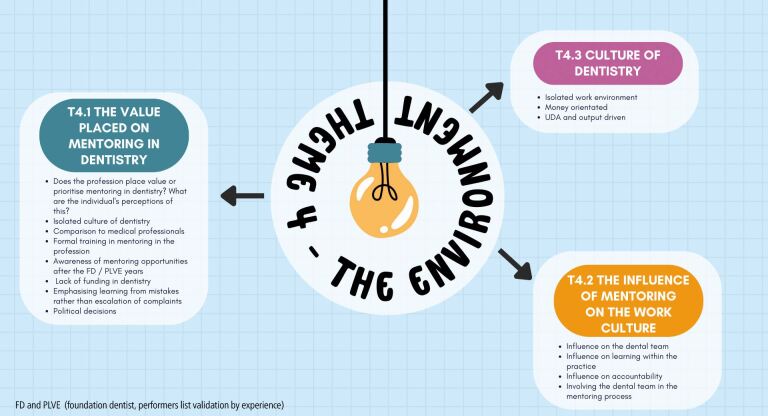
Theme 4: the environment for mentorship

The final theme is illustrated in Figure 4.

There was a general consensus among the participants that while they as individuals placed importance on the value of mentoring in dentistry, the profession itself did not:

- ‘*I don't think they [the wider profession] put a lot of value on it [mentoring] actually'* P2.

One participant spoke of conflicting views around mentoring within the profession:

- ‘*I do think in certain circles [mentoring is valued] very highly. I think on the flip side of that, in certain circles there are people that just sort of don't understand its needs…*' P4.

Participants spoke about the potential influence mentoring could have on the work culture:

- *‘…I don't know how much it would lead to retention, but maybe recruitment it would make people more confident to take on slightly more complicated cases…'* P1
- ‘*There's more of an environment of learning and development than perhaps there would have [been]*' P7.

Others pointed out the risk of creating a mentoring environment which may undermine the mentee's own confidence by reinforcing a hierarchy:

- ‘*So at times it can create a culture where they're [mentees are] not trusted to have their own opinions…it's not supporting them in their journey…*' P6.

## Discussion

Participants viewed mentoring positively in helping dealing with complaints, reducing anxiety and increasing confidence. These findings align with Moore *et al.,*^11^ that mentoring programmes have the potential to positively influence individuals. Results also showed that participants valued mentoring: ‘*a lot [of value]. I think it [mentoring] can define your career really'* (P7), supported by Mei *et al.,*^10^ who found that mentorship influenced the short-term career plans of individuals.

However, the results reported here found that mentoring did not change participants' perceptions of their job either positively or negatively, contradicting Moore *et al.*'s^11^ proposition. Participants suggested that mentoring could lead to a reduction in the feeling of isolation, which confirms the findings of Leadbeatter *et al.*^12^ in terms of creating a sense of belonging.

The majority spoke of peers' negative experiences, stating the influence that mentorship could have on individuals and their perception of their job/profession. This highlighted the variation of experiences of mentoring in dentistry, as well as the potential negative consequences where adequate support was lacking. When discussing negative mentoring experiences, it was noted that participants attributed these to peers. Participants recounted those experiences indirectly, presenting them as experiences of others rather than claiming them as their own. This distancing may reflect a reluctance to self-identify with negative experiences, possibly to maintain a positive self-image or avoid potential repercussions in professional contexts. Further research is needed to explore negative mentoring experiences, perhaps using alternative data collection tools to allow anonymity for participants to be able to disclose those experiences.

The diversity of positive and negative experiences begs two questions: does the delivery of mentoring in dentistry need to be reassessed with a particular focus on the selection of mentors? Secondly, to what extent can negative experiences influence an individual's career and mental health?

### Perception of an ideal mentorship

The results found that participants experienced mentoring during their FD year, or PLVE, or after this term. The majority of participants declared that they had little expectation before commencing their mentoring experience. However, one participant (P6) stated that they were given the opportunity to have discussions with all the potential mentors in their scheme and both mentors and mentees were able to rank one other based on their preference and therefore knew what to expect. Kibbe *et al.*^15^ argued that training mentees in how to choose a mentor and allowing mentees to choose their mentors could improve the mentoring experience and make them more invested in the mentoring relationship. However, the expectation of mentors often overlapped with having a teacher, who physically demonstrated how things should be done; although, participants' views were in line with general characteristics of mentors.^16^

### Perceived value of mentorship within the profession

Participants' perception of the value of mentorship within dentistry was low, with participants stating a lack of funding, support and formal training for mentors to be contributing factors. This suggests that if mentoring was a requirement from governing bodies, such as the General Dental Council or the NHS, then it would be seen as being valued in the profession. Additionally, mentorship within the profession and outside of the FD/PLVE year only surfaces when concerns are raised about practitioners. Dentists are advised to seek help for remedial action to reassure members scrutinising their work of their competencies.^26^ It could be argued that this in itself positions mentoring as something which is only sought out of necessity rather than a tool which seeks to develop and support individuals. If mentoring were to become an integral requirement and part of professional development, then its perceived value would increase within the profession.

Participants expressed the difficulty in measuring and quantifying the value of mentoring which is in line with Grant *et al.*'s^14^ argument. However, a quantifiable financial benefit of mentoring could be FDs being retained by the dental practice following the completion of their FD year, as suggested by McKimm *et al.*^8^ Going forward, instead of seeking the value of mentoring in a financial sense, the influence of mentoring on retention and recruitment could be explored. It may be difficult to find a direct causal relationship between mentoring and retention; however, research in this field could add to the argument for creating and establishing a mentoring culture.

## Conclusion

Results from this qualitative study demonstrated how mentorship is valued for both professional and personal influence on early career practitioners. Wider challenges about how mentorship is viewed within the profession in general were also highlighted. The results presented in this study are not a representation of all early career dentists and rather provides a deep insight into this group of participants and their views around mentorship experiences which can apply to others within the profession.^27^^,^^28^ The data also highlighted the need for a wider debate about the role of mentorship in staff retention within NHS dentistry^2^ and for support for wellbeing.

Future research should focus on expanding participation to more experienced practitioners and how they view their mentorship experiences, how they seek support, and how they offer mentorship support to others.

The recommendation is for mentoring schemes to ensure appropriate training for mentors, recognising that formal mentorship is required after the foundation year. Creating a supportive and flourishing culture within the workplace for mentoring interactions is also needed with a particular focus on developing individuals and addressing the isolation of dental professionals.

## Data Availability

Data are available from the corresponding author upon reasonable request and in line with institutional data retention and ethical guidelines.

## References

[1] Standing Committee on Postgraduate Medical and Dental Education. *Supporting Doctors and Dentists at Work: An Enquiry into Mentoring.* 1998.

[2] NHS. Interim NHS People Plan. 2019. Available at https://www.england.nhs.uk/wp-content/uploads/2022/07/Interim-NHS-People-Plan_June2019.pdf (accessed 1 January 2026).

[3] Health Education England. HEE's Advancing Dental Care Review: Final Report. 2021. Available at https://www.hee.nhs.uk/sites/default/files/documents/Advancing%20Dental%20Care%20Report%20Sept%2021.pdf (accessed 1 January 2026).

[4] British Dental Association. Nearly half of dentists severing ties with NHS as government fails to move forward on reform. 2022. Available at https://bda.org/news-centre/press-releases/Pages/nearly-half-of-dentists-severing-ties-with-nhs.aspx (accessed 25 August 2024).

[5] Dental Protection. Breaking the burnout cycle. 2019. Available at https://www.dentalprotection.org/docs/dentalprotectioninternationallibraries/dpl-publications/ireland/1907310561-ire-dp-burnout-policy-paper.pdf (accessed 25 August 2023).

[6] Ng K Y B, Lynch S, Kelly J, Mba O. Medical students' experiences of the benefits and influences regarding a placement mentoring programme preparing them for future practice as junior doctors: a qualitative study. *BMJ Open* 2020; DOI: 10.1136/bmjopen-2019-032643. 10.1136/bmjopen-2019-032643 PMC7044925 31941766

[7] Wynn S, Holden C, Romero S, Julian P. The importance of mentoring in nursing academia. *Open J Nurs* 2021; **11:** 241–248.

[8] McKimm J, Jolie C, Hatter M. *Mentoring: Theory and Practice*. *Preparedness to Practice Project, Mentoring Scheme.* 2007.

[9] Schwartz B, Saad M N, Goldberg D. Evaluating the students' perspectives of a clinic mentoring programme. *Eur J Dent Educ* 2014; **18:** 115–120. 10.1111/eje.12065 24118706

[10] Mei L, Lai Y, Lee P *et al.* Final year dental students' career plans, work patterns, work-life balance and domestic life in New Zealand and Australia. *Eur J Dent Educ* 2020; **24:** 679–686. 10.1111/eje.12556 32537849

[11] Moore R, Molsign S, Meyer N, Schepler M. Early clinical experience and mentoring of young dental students – a qualitative study. *Dent J (Basel)* 2021; **9:** 91. 10.3390/dj9080091 PMC8392303 34436003

[12] Leadbeatter D, Madden J, Ross B, Russell E. Transition to dental practice: newly graduated dentists' views of being successful in dental practice. *Eur J Dent Educ* 2020; **24:** 753–762. 10.1111/eje.12565 32593181

[13] Dahlberg M L, Byars-Winston A. Assessment and evaluation: what can be measured in mentorship, and how? *In* Dahlberg M L, Byars-Winston A (eds) *The Science of Effective Mentorship in STEMM*. Washington DC: National Academies Press, 2019. 31958221

[14] Grant A M. ROI is a poor measure of coaching success: towards a more holistic approach using a well-being and engagement framework. *Coaching An Int J Theory Res Pract* 2012; DOI: 10.1080/17521882.2012.672438.

[15] Kibbe M R, Pellegrini C A, Townsend C M Jr, Helenowski I B, Patti M G. Characterization of mentorship programs in departments of surgery in the United States. *JAMA Surg* 2016; **15:** 900–906. 10.1001/jamasurg.2016.1670 27383863

[16] Schrubbe K F. Mentorship: a critical component for professional growth and academic success. *J Dent Educ* 2004; **68:** 324–328. 15038633

[17] Royal College of Surgeons of England. Toolkit for surgical mentorship. Available at https://diversity.rcseng.ac.uk/develop-and-learn/mentorship/ (accessed 30 August 2023).

[18] Holt V P, Ladwa R. Mentoring. A quality assurance tool for dentists. Part 3: building a successful mentoring relationship. *Prim Dent Care* 2009; **16:** 67–73. 10.1308/135576109787909355 19366522

[19] Parloe E, Wray M J. *Coaching and Mentoring: Practical Methods to Improve Learning*. p 183. London: Kogan Page, 2000.

[20] Bowling A. *Research Methods in Health: Investigating Health and Health Services*. 4^th^ ed. Maidenhead: Open University Press, 2014.

[21] Kuper A, Lingard L, Levinson W. Critically appraising qualitative research. *BMJ* 2008; DOI: 10.1136/bmj.a1035. 10.1136/bmj.a1035 18687726

[22] Holloway I, Wheeler S. *Qualitative Research in Nursing and Healthcare*. 3^rd^ ed. Chichester: Wiley-Blackwell, 2010.

[23] Ritchie J, Lewis J, Nicholls C M, Ormston R. *Qualitative Research Practice: A Guide for Social Science Students and Researchers*. pp 52–54. 2^nd^ ed. London: Sage, 2014.

[24] Silverman D. *Interpreting Qualitative Research: Methods for Analysing Talk, Text and Interaction*. 4^th^ ed. p 274. London: Sage, 2011.

[25] Braun V, Clarke V. Using thematic analysis in psychology. *Qual Res Psychol* 2006; **3:** 77–101.

[26] General Dental Council. Evaluation of remediation support in UK dentistry. 2015. Available at https://www.gdc-uk.org/docs/default-source/research/evaluation-of-remediation-support-in-dentistry-report-final-may-2015.pdf?sfvrsn=b643896f_2 (accessed 1 January 2026).

[27] Noble H, Smith J. Issues of validity and reliability in qualitative research. *Evid Based Nurs* 2015; **18:** 34–35. 10.1136/eb-2015-102054 25653237

[28] Nathwani S, Rahman N. GROWing in dentistry: mentoring the dental professional. *BDJ Team* 2022; **9:** 20–26. 10.1038/s41415-022-3979-2 PMC8874297 35217747

